# Effects of Platycodin D on Reflux Esophagitis due to Modulation of Antioxidant Defense Systems

**DOI:** 10.1155/2018/7918034

**Published:** 2018-03-26

**Authors:** Su-Yeon Cho, Chang-Hyun Song, Ji-Eun Lee, Seong Hun Choi, Sae-Kwang Ku, Soo-Jin Park

**Affiliations:** ^1^Medical Products Safety Division, Daejeon Regional Food and Drug Administration, Ministry of Food and Drug Safety, Daejeon, Republic of Korea; ^2^Department of Histology and Anatomy, College of Korean Medicine, Daegu Haany University, Gyeongsan, Republic of Korea; ^3^The Medical Research Center for Globalization of Herbal Formulation, Daegu Haany University, Gyeongsan, Republic of Korea

## Abstract

**Aims:**

The effects of platycodin D (PD) pretreatment were examined in reflux esophagitis (RE) induced rats.

**Methods:**

Sham, control, and omeprazole (OMP) group were pretreated with distilled water or OMP as a reference, respectively, and PD pretreated groups were given 3 different PD doses once a day for 7 days. One hour after last pretreatment, RE was induced by ligation of the forestomach and pylorus. At 8 h after operation, all animals were sacrificed.

**Results:**

PD showed significant dose-dependent reduction of gastric secretion, myeloperoxidase activity, and RE lesion areas of esophagus and stomach mucosa. There was a reduction of lipid peroxidation in 2 doses of PD groups and elevation of antioxidant enzyme activity in all PD groups. Gastric hexose and sialic acid were significantly increased in PD groups, while collagen was reduced. Plasma histamine levels were significantly reduced in all PD groups, but not in the OMP group. Total invasive lesion sizes of esophagus and gastric fundus were significantly decreased in all PD groups. Thicknesses in esophagus of all PD groups were significantly decreased and thicknesses of funds were significantly increased except lowest PD dose.

**Conclusions:**

Therapeutic effects of PD on the esophageal and gastric lesions were shown in RE induced rats dose-dependently. The PD pretreatment had significant antioxidant effects with regulation of histamine levels. This study provides useful information regarding the effectiveness of the drug for RE and further novel drug discovery using natural herbal products.

## 1. Introduction

Reflux esophagitis (RE) is an esophageal mucosal injury and is caused by reduced motility in the lower esophageal sphincter, leading to an abnormal increase in gastric contents [1]. The abnormal reflux of gastric contents typically damages the distal esophagus and gastroesophageal junction, inducing inflammatory responses in the esophageal mucosa. The general RE symptoms are regurgitation, dysphagia, and heartburn, but chronic RE can induce severe complications including laryngitis, esophageal stricture, and Barrett's esophagus and may eventually lead to cancer [2]. RE is a common digestive tract disorder in Western countries with a relatively high prevalence of 10–30% of individuals reporting weekly symptoms [3] and is also increasingly common in Korea, probably due to irregularities in lifestyle, obesity, and diet-induced gastric irritation by alcohol, caffeine, or aspirin [4]. Recent studies have shown a strong connection between the pathogenesis of RE and oxygen-derived free radicals and that oxidative stress has important roles in RE pathogenesis [5, 6].

Current RE treatments can be categorized according to the following mechanisms: antacids, H_2_ receptor antagonists, proton pump inhibitors (PPI), and gastric motility agents. Although many drugs have been developed to treat RE, more than 40–60% of patients still suffer from stricture or other complications including cancers and many remain far from full recovery despite adequate treatment time with acid secretion suppressants [7]. Therefore, effective therapeutic strategies remain in the public interest, thereby underlining the need for further drug discovery.

Attention towards natural products as novel drug materials has been increasing in the drug development area.* Platycodon grandiflorum*, also known as platycodi radix, are commonplace plants in Korea, Japan, and China, and the root is used as a food and herbal medicine for preventing flu, cough, metabolic disorders, stomachache, diarrhea, postnatal illness, insomnia, and infection. In particular, the root is known to contain a large quantity of saponin, which is known to consist of platycodin A, C, and D, as well as polygalacin D, spinasterol, spinasterol glucoside, inulin, and so on [8, 9]. Platycodin D (PD) is a major medicinal element of* Platycodon grandiflorum* [9] and has also been shown to have anticancer [10, 11], anti-inflammatory [12, 13], antiobesity [14], antidiabetes [15], cholesterol-lowering [16], and antinociceptive [17] properties as well as immunoregulatory effects [18]. PD also affords protection against oxidative stress which is useful in treating various diseases [19, 20]. Therefore, the therapeutic effects of PD on esophageal and stomach mucosal injuries were examined in RE induced rats in this study.

## 2. Materials and Methods

### 2.1. Animals

All experiments were conducted after attaining approval of protocol by Institutional Animal Care and Use Committee (IACUC) at Daegu Haany University (Gyeongsan, Korea; IACUC number DHU013-097, December 24, 2013). Male Sprague-Dawley rats (6 weeks, Japan SLC Inc, Shizuoka, Japan) were maintained at 20–25°C and humidity of 40–45% with 12 h light/dark cycle. Water and normal rodent pellet diet were supplied* ad libitum*.

### 2.2. Materials Preparation and Pretreatments

PD, a bidesmoside triterpenoid consisting of an aglycone moiety, 3-Glc, and 28-O-Api-Xyl-Rha-Ara, was extracted from raw platycodin radix by previous method [16] and provided from Glucan Corp. Ltd (Busan, Korea). The PD was identified by comparing to confirmed PD sample based on radio frequency (Rf), fast atom bombardment mass spectrometry (FAB-MS; = 1225.38), and [^13^C]-NMR spectra. The purified PD was in light yellow powder form and stored in a desiccator at 4°C until use. PD was dissolved to at least 20 mg/ml in distilled water and used at 3 different doses (200, 100, or 50 mg/kg). Omeprazole (OMP) (Sigma, MO, USA) was dissolved in distilled water and used at a dose of 10 mg/kg as a reference drug [21]. The OMP was kept in a desiccator at room temperature before use. After acclimatization for 1 week, rats were allocated into six groups of 8 rats with random manner. OMP, PD 200, PD 100, and PD 50 groups were pretreated with omeprazole 10 mg/kg, PD 200 mg/kg, 100 mg/kg, and 50 mg/kg once a day for a week, respectively. Sham and control group were orally administered distilled water once a day for a week.

### 2.3. Induction of RE and Sampling

**Figure 1:**
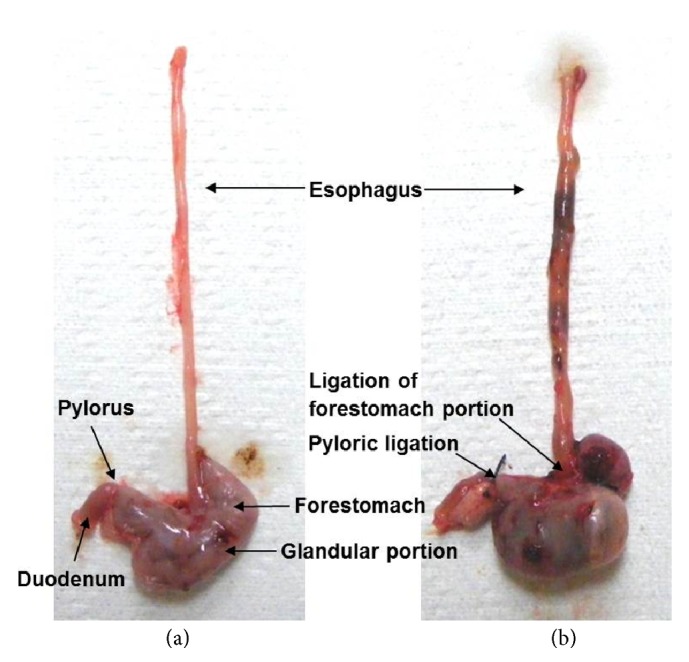
Sham surgery (a) or reflux esophagitis induction surgery by ligation of the forestomach and pylorus (b).

Sham and the other groups received a sham surgery or RE induction surgery 1 h after the last pretreatment, respectively [22]. Rats were anesthetized with Zoletil mixture™ (25 mg/kg, i.p.; Virbac, France). For RE induction, the abdomen was opened by a 2 cm median incision. The transitional region between the forestomach and corpus and the pylorus portion were ligated with a 2–0 silk thread (B. Braun Surgical SA, Spain) (Figure 1). A 1 cm longitudinal cardiomyotomy was performed across the gastroesophageal junction. The incised region was immediately sutured. For the sham, the abdomen was opened in the same manner as the RE induction and closed immediately without ligation and cardiomyotomy. All animals were sacrificed after collection of 1 ml of blood from the orbital plexus at 8 h after surgery and samples of esophagus and stomach tissue; gastric contents were collected.

### 2.4. Measurements of Body Weights Changes

Body weight was measured 1 day before pretreatment and at days 0, 1, 5, and 6 after pretreatment using an automated electronic balance (Precisa Instrument, Switzerland). Body weight gains were calculated according to the following equation:

(1) Body  weight  changesg=body  weight  at  sacrificeday  6−body  weight  at  initial  pretreatmentday  0.

### 2.5. Measurements of Gross Lesions

Tissue samples of esophagus and stomach were fixed in 10% neutral buffered formalin. Gross lesions of esophagus or stomach were measured as a total area (mm^2^) with a light microscope using a 4x objective lens (Nikon, Japan) and scored according to previous method [22].

### 2.6. Measurements of Gastric Volume and Contents

The gastric contents were centrifuged for 5 min at 2000 ×g, and the volume of the supernatant was described as ml/kg of body weight. Titration of 0.01 N NaOH was done for estimation of acid level using phenolphthalein as an indicator [23]. Level of Pepsin was determined using hemoglobin as a substrate [24].

### 2.7. Esophageal Myeloperoxidase (MPO) Activity

About 200 mg of esophageal samples were homogenized in 10 volumes of cold potassium phosphate buffer (50 mM K_2_HPO_4_, pH 6.0; Sigma, MO, USA) with hexadecyltrimethyl-ammonium bromide (HETAB; 0.5% w/v; Sigma, MO, USA). After centrifugation at 12,000 ×g and 4°C for 10 min, the supernatant was removed and rehomogenization of remains was done with an equivalent volume of 50 mM K_2_HPO_4_ containing 0.5% (w/v) HETAB and 10 mM EDTA (Sigma, MO, USA). The measurement of H_2_O_2_-dependent oxidation of* o*-dianizidine·2 HCl was performed for estimation of MPO activity. MPO levels/g of tissue weight that caused a 1.0/min change in absorbance at 460 nm and 37°C was expressed as one unit (U) of enzyme activity [22].

### 2.8. Measurement of Antioxidative Defense System

Tissue of Fundus was homogenized in cold 0.01 M Tris-HCl buffer (pH 7.4). After centrifugation of homogenate at 800 ×g for 10 min, the supernatant was centrifuged at 12,000 ×g for 15 min to obtain mitochondrial fraction [25]. Esophageal protein contents were estimated by the Lowry method [26]. Concentration of malondialdehyde (MDA), an indicator of lipid peroxidation (LPO), [27] was determined by the Jamall and Smith's method [28]. The measurement of inhibition of a nicotinamide adenine dinucleotide (reduced)-phenazine methosulphate-nitroblue tetrazolium reaction system was conducted for estimation of superoxide dismutase (SOD) activity [29]. One U of SOD activity, expressed as U/mg protein, is equivalent to 50% inhibition of the formazan structure for 1 min at room temperature. The decomposition of H_2_O_2_ in the presence of CAT was measured at 240 nm [30]. One U of CAT was defined as the amount of enzyme for decomposing 1 *μ*mol of H_2_O_2_/min at 25°C and pH 7.0. Glutathione (GSH) content was estimated by the method described previously [31].

### 2.9. Measurement of Gastric Mucosal Component

Total hexose was analyzed by the reaction of carbohydrate in concentrated sulfuric acid with 5-methyl orcinol and measured colorimetrically by previous method [32]. The total hexose was determined according to a standard graph plotted using galactose-mannose. Protein binding of sialic acid was estimated by a thiobarbituric acid assay [33]. After treating esophageal homogenate with 90% ethanol, the precipitate was dissolved in 0.2 N sulfuric acid and oxidized with periodic acid and incubated at 37°C for 30 s. Oxidation was terminated with sodium arsenate and cyclohexane and 6% thiobarbituric acid were added. The mixture was centrifuged to obtain a clear pink layer of cyclohexane, and the color intensity was observed at 550 nm. For the measurement of collagen content, the esophageal mucosa was hydrolyzed in 6 mol/l HCl at 110°C for 18 h. The evaporations of acid hydrolysates were conducted in a heat block at 95°C. With 1.0 ml deionized water, the dried residues were rinsed three times with complete evaporation between each wash step. The acid-free samples were suspended again in 1.0 ml acetate-citrate buffer and sonicated for 30 min [34].

### 2.10. Blood Histamine Level Detection

The plasma samples were treated with 0.2 M perchloric acid and centrifuged at 10,000 ×g and 4°C for 30 min. The supernatant was used for the determination of plasma histamine levels by high performance liquid chromatography [35].

### 2.11. Histopathological Processing and Analysis of the Esophagus and Fundus of Stomach

Individual esophagi were fixed in 10% neutral buffered formalin, and esophageal regions from approximately 5 cm above the esophageal-cardiac junction and the fundus were prepared for histopathological analysis as described previously [22]. The tissue was trimmed crosswise based on the lumen. All trimmed esophagi and fundi were fixed in 10% neutral buffered formalin. 3-4 *μ*m sections were prepared after paraffin embedding. Hematoxylin and eosin (H&E) stain were performed for staining of typical sections and histological profiles were determined. The total thicknesses of esophageal and fundic walls were defined as thickness from luminal mucosal surface to tunica adventitia of esophagus or serosa of fundus. They were measured in the cross-trimmed histological specimens using a digital image analyzer (DMI-300, DMI, Korea). In addition, invasive lesions of esophagus and stomach were estimated using a lesion length and total thickness across the organ wall. Lesion invasiveness (%) was calculated according to the following equation [22]:

(2) $$\begin{array}{c} \text{Lesion invasiveness}\left(\;\%\;\right)=\left(\;\frac{\text{Length of lesions in the cross-trimmed esophageal or fundic walls}}{\text{total thickness of cross-trimmed esophageal or fundic walls}}\;\right)\times 100. \end{array}$$

### 2.12. Statistical Analyses

Numerical data are presented as means ± SD According to results of Levene test, when the results showed no significant deviation in variance homogeneity, a one-way analysis of variance (ANOVA) and a least significant difference (LSD) test were done. If there were significant deviations in variance homogeneity, the Kruskal-Wallis *H* test and Mann–Whitney *U* (MW) test were conducted. Statistical software was SPSS for Windows release 14.0 K, (SPSS Inc., Chicago, IL, USA), and *P value *< 0.05 was considered of statistical significance. In addition, the percent changes compared to sham (PCS) were calculated to estimate the severities of esophageal-fundus damage induced by the RE induction surgery according to (3), and the percent changes compared to control (PCC) were calculated to assess the efficacy of the PD or OMP according to (4):

(3) PCS%=Data  from  control−Data  from  shamData  from  sham×100,

(4) PCC%=Data  from  PD  or  OMP  pretreated  rats−Data  from  controlData  from  control×100.

## 3. Results

### 3.1. Body Weight Changes and Gross Anatomical Features of the Esophagus and Stomach

There were no differences in body weight among the groups over the course of the 7-day pretreatments (Table 1).

**Figure 2:**
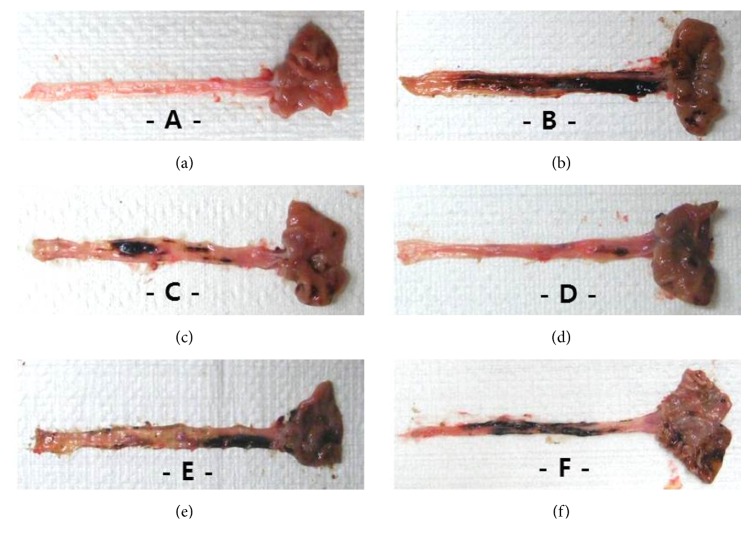
Macroscopic appearance of esophageal and gastric mucosa in pretreatment groups. Panels: (a) sham, (b) control, (c) OMP, (d) PD 200, (e) PD 100, and (f): PD50. Compared to sham (a), severe focal lesions with hemorrhage and ulcer exhibited in the esophageal and gastric mucosa of control (b). However, the macroscopic lesions were dose-dependently reduced by treatment with OMP (c) or PD (d, e, f). PD: platycodin D, OMP: omeprazole.

Gross changes were examined in the esophagus and gastric mucosa. Diffused lesions were observed in control compared to sham along with large areas of hemorrhage and ulcer after surgery. However, the size and severity of lesions seemed to be reduced in OMP and all PD groups compared to control (Figure 2).

**Figure 3:**
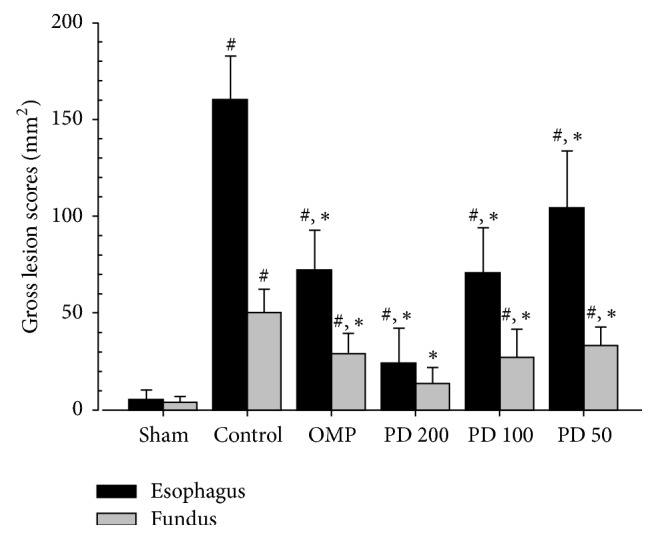
Effects of platycodin D on lesion area in the esophageal and gastric mucosa. Values are expressed as mean ± SD of eight rats; PD: platycodin D, OMP: omeprazole. ^#^*P* < 0.01 compared with sham; ^*∗*^*P* < 0.01 compared with control.

The mucosal lesion areas were significantly increased in control compared to sham (*P* < 0.01), indicating a suitable induction of RE. The PCS of actual lesion of esophagus and gastric mucosa was 2840.8% and 1115.3%. Seven-day pretreatment with PD significantly reduced lesion areas compared to control (*P* < 0.01). The PCC of esophagus lesion areas in PD 200, PD 100, PD 50, and OMP were −84.9%, −55.8%, −34.8%, and −54.9% and those of gastric lesion areas in PD 200, PD 100, PD 50, and OMP were −73.1%, −46.1%, −33.4%, and −42.2% %, respectively (Figure 3).

### 3.2. Gastric Secretion in the RE Induced Rats

Gastric volume and levels of gastric acid and pepsin were significantly reduced in PD groups compared to control (*P* < 0.01) (Table 2).

### 3.3. Analyses of Esophageal Damage

**Figure 4:**
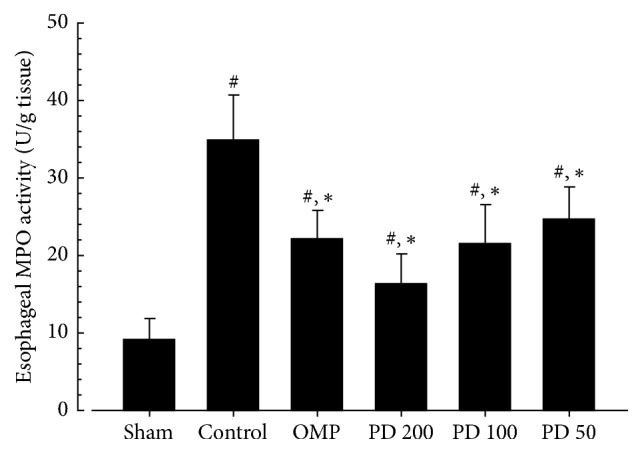
Effects of platycodin D on myeloperoxidase activity. Values are expressed as mean ± SD of eight rats; ^#^*P* < 0.01 compared with sham; ^*∗*^*P* < 0.01 compared with control. PD: platycodin D, OMP: omeprazole.

The activity of MPO was significantly increased in control compared with sham (*P* < 0.01) and the PCS of MPO activity was 283.7%, showing granulocyte accumulation after esophageal damage. However, pretreatment with PD significantly reduced esophageal MPO activity compared to control (*P* < 0.01). The PCC of MPO in PD 200, PD 100, PD 50, and OMP was −53.3%, −38.1%, −29.4%, and −36.6%, respectively (Figure 4).

### 3.4. Analyses of Antioxidant Defense Systems

The LPO of control significantly increased compared to sham (237.2%, *P* < 0.01), reflecting increased cell membrane damage and oxidation by RE induction. LPO was significantly reduced in PD groups compared to control except PD 50. The stomach SOD activity of control was decreased compared to sham (−53.6%, *P* < 0.01) and SOD was significantly increased in PD 200 (*P* < 0.01), PD 100, and PD 50 (*P* < 0.05) compared to control. CAT of control was reduced compared to sham (−57.2%, *P* < 0.01). However, the CAT was significantly increased in PD 200 (*P* < 0.01) and in PD 100 and PD 50 (*P* < 0.05) compared to control. GSH was reduced in control as compared to sham (−59.5%, *P* < 0.01) and significantly increased in PD 200, PD 100 (*P* < 0.01), and PD 50 (*P* < 0.05) (Table 3).

### 3.5. Analyses of Gastric Mucosal Components

The total hexose and sialic acid of control were reduced compared to sham, respectively (−49.9%, −49.5%, *P* < 0.01). However, they were significantly increased in all PD groups as well as in the OMP group compared to control (*P* < 0.01). PCS of collagen levels was 128.7% (*P* < 0.01). However, it was significantly reduced in all PD groups compared to control (*P* < 0.01) (Table 4).

### 3.6. Estimation of Plasma Histamine Levels

**Figure 5:**
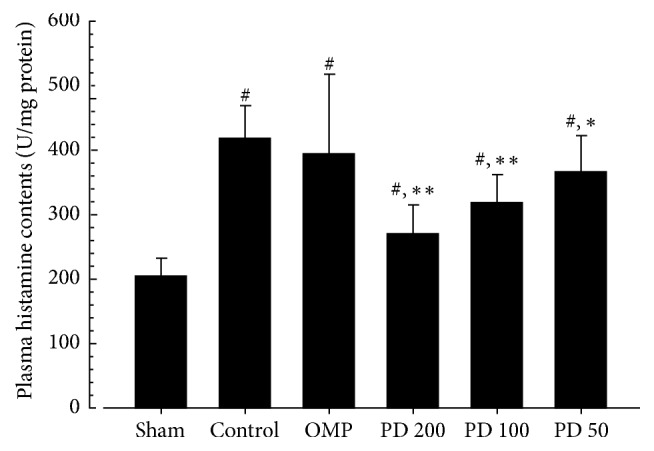
Effects of platycodin D on levels of plasma histamine. Values are expressed as mean ± SD of eight rats. PD: platycodin D, OMP: omeprazole. ^#^*P* < 0.01 compared with sham; ^*∗*^*P* < 0.05 and ^*∗∗*^*P* < 0.01 compared with control.

Compared to sham, histamine levels appeared to be increased in control (104.7%, *P* < 0.01). It was significantly decreased in PD 200, PD 100 (*P* < 0.01), and PD 50 (*P* < 0.05) compared to control. However, there was no significant changes in OMP as compared to control (Figure 5). The PCC of histamine levels in PD 200, PD 100, PD 50, and OMP were −35.7%, −24.0%, −12.6%, and −5.8%, respectively.

### 3.7. Histopathological Analyses of the Esophagus and Fundus of Stomach

**Figure 6:**
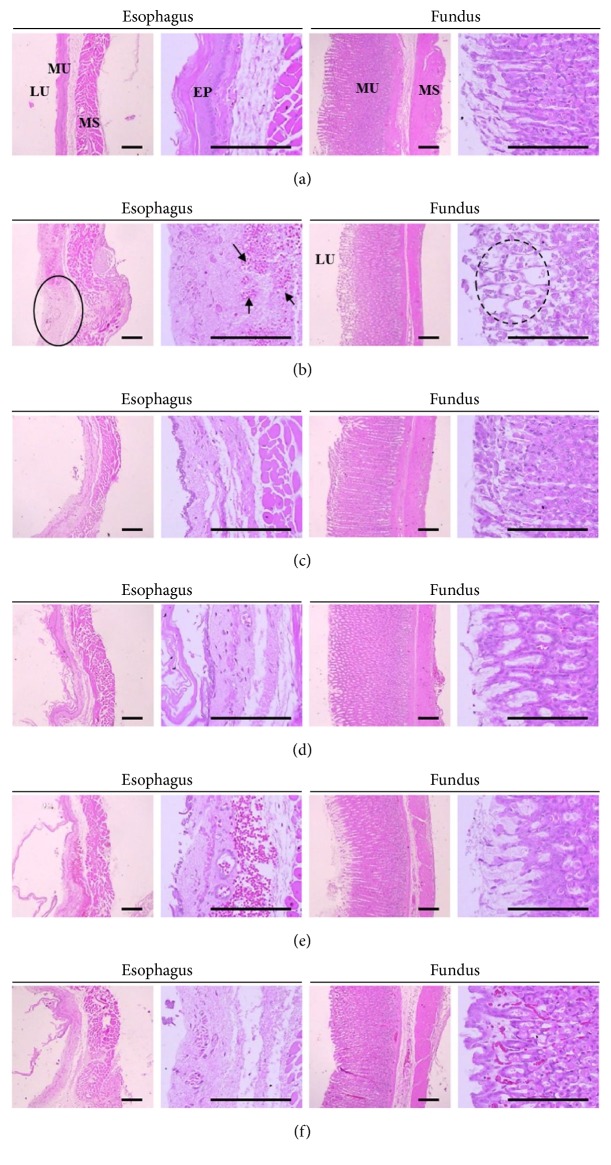
Histopathological analyses of invasive lesions and total organ wall thickness. The panels show representative histopathological profiles of a hematoxylin-eosin stain of the esophagus and gastric fundus in sham or RE induced rats. Panels: (a) sham, (b) control, (c) OMP, (d) PD 200, (e) PD 100, and (f) PD50. Histopathological analysis showed severe focal lesions with hemorrhage (black arrow), ulcer (circle), and edematous changes (dashed line circle) in the esophagus and gastric fundus of control compared with sham. However, the lesions were notably reduced by treatment with each of the 3 doses of PD compared with control; PD: platycodin D; OMP: omeprazole; LU: lumen; EP: epithelium; MU: mucosa; MS: muscular layer; Scale bars = 80 *μ*m.

Histopathological assessment showed diffused ulcer and mucosal hyperplasia with hemorrhage and edema in the esophagus and gastric fundus in control (Figure 6(b)). However, these lesions seemed to be milder after the 7-day pretreatment with either PD or OMP as compared to control (Figures 6(c)–6(f)).

Invasive lesion size and total thickness of esophagus were notably increased in control compared to sham (*P* < 0.01). As shown in Table 5, the invasive lesion size was significantly reduced in the PD 200, PD 100, OMP (*P* < 0.01), and PD 50 (*P* < 0.05) compared to control. In addition, total thickness was reduced in all PD groups (*P* < 0.01). Compared to sham, invasive lesion size of fundus of control was increased (*P* < 0.01). However, the PD and OMP pretreatment groups showed significant reductions in invasive lesions compared to control (*P* < 0.01). Similarly, total thickness was significantly decreased in control compared to sham (*P* < 0.01). The PD 200, PD 100, and OMP pretreatment groups showed a significant increase in the total thickness compared to control (*P* < 0.01) (Table 5).

## 4. Discussion

The* Platycodon *root is a natural product that has a long history of widespread use in Korean herbal medicine as an antiphlogistic, antitussive, and expectorant agent. The chemical components of platycodin are relatively well-known [8, 9], and a single PD compound isolated from the root of* Platycodon grandiflorum *is available as a commercial dietary supplement for a variety of medicinal applications, such as flu, cough, metabolic disorders, stomachache, diarrhea, and inflammatory infection. In particular, the anti-inflammatory activity of PD has been shown in animals [13, 36]. In this experiment, 3 different doses of PD were given as pretreatment for 7 days to RE induced rats and the therapeutic effects were compared with a reference drug, OMP, which is one of the most widely used medicines for RE.

*Therapeutic Effects of PD on RE.* Like the findings of other studies [37–39], PD reduced mucosal inflammation in the esophagus and stomach. The biochemical data revealed reduction of MPO by inhibition of neutrophil infiltration into the injured mucus of the RE, and a reduction of histamine levels by the inhibition of mast cells or basophils in inflammatory pathways (Figures 4 and 5). Although collagen fibers provide structural integrity for tissues under normal conditions, impaired collagen fibers during RE induce sclerosis with stricture formation in the esophagus [40]. Esophageal collagen levels were reduced in PD groups as well as in the OMP group compared to control (Table 4). The anti-inflammatory effects of PD resulted in an improvement in the lesions associated with mucosal injuries, showing a reduction in gross lesion area (Figures 2 and 3) and gastric contents including acids and pepsin (Table 2). PD treatment was also associated with increases in the levels of gastric mucosa components, total hexose and sialic acids (Table 4), and milder histopathological lesions (Table 5 and Figure 6). Gastric contents, including acids and pepsin secreted from parietal cells, are a potentially damaging factor in the esophageal and gastric mucosa. The reduced histamine levels may inhibit the secretion of gastric acids from the parietal cells that normally occurs in response to histamine and reduce damage to the mucosal membranes [41, 42]. Therefore, these findings suggest that PD has therapeutic potential for gastroesophageal reflux disease.

*Antioxidative Effects of PD on RE.* The pathogenesis of RE has multiple factors, particularly since the gastric contents refluxing into the esophagus contain a variety of components [43, 44]. Although exposure of the esophageal membranes to gastric acids influences the severity of RE, oxidative stress also plays an important role in mucosal erosion as well as the secondary damage to the mucosal layer by mechanical digestive movement [6]. In present study, LPO, SOD, CAT, and GSH were used to prove for antioxidative effects of PD. Increased production of oxygen-derived free radicals is accompanied by enhanced mucosal lipid peroxidation, which is used as an indicator of mucosal membrane damage caused by free radicals [5]. The control showed highly increased LPO (3.4-fold) as compared to sham. However, LPO was significantly reduced in the PD 200 and PD 100 pretreatment groups (Table 3), suggesting inhibition of oxygen-derived free radical production by treatment with higher doses of PD. This suggests that PD inhibits oxidative stress by enhancing antioxidant enzymes, which is supported by the increased levels of SOD, CAT, and GSH in all PD groups (Table 3). Catalase (CAT) activity is represented for capacity to degrade hydrogen peroxide which produces hypochlorous acid and the highly toxic hydroxyl free radical [45]. Glutathione (GSH) is involved in preventing oxygen-derived free radicals from contributing to the pathogenesis of RE [46, 47].

*Relevance of Antioxidant Effects for RE Therapeutics.* Since the esophageal and gastric mucosa are injured not only by gastric acids alone but also by oxidative stress and oxygen-derived free radicals, therapeutic strategies also focus on antioxidants as well as regulating pH. One approach may involve enhancement of the natural antioxidative mechanisms in the body, for example, upregulation of antioxidative enzymes such as SOD, CAT, or GSH. There has been extensive research into natural herbal-derived compounds for combating oxidative stress and free radical damage [48]. A 7-day pretreatment with PD enhanced antioxidative pathways in RE induced rats suggests that PD can function as a free radical scavenger that can oppose oxidative stress. Another approach is treatment with exogenous antioxidants. Thiol-containing GSH, a nonenzyme antioxidant, promotes the detoxification of several toxic metabolites and exogenous thiol compounds have been shown to protect the stomach from ethanol-induced injuries [47]. An acidic environment and histamine release can both induce degradation of mucosal glycoprotein [46, 49]. In addition, high levels of histamine secretion have been suggested to be involved in the pathogenesis of gastric ulceration, inflammation, esophagitis, and gastric acid secretion [42]. The antioxidant effects of PD may be involved in the observed reduction in histamine levels, leading to the increased levels of glycoproteins (Table 4). Indeed, the histopathological lesions associated with the experimental RE used here appeared to be ameliorated by PD treatment (Figure 6). OMP showed similar effects with PD except on histamine levels, which is in agreement with other studies [23, 50]. It is probably due to the function of OMP as a PPI involved in reducing gastric acids by inhibition of proton pump rather than inhibiting oxidative stress. Therefore, PD may have a therapeutic efficacy similar to or better than OMP in human patients with RE.

*Other Mechanisms Involved in the Therapeutic Effects of PD.* The therapeutic effects of PD on RE may result from immunomodulatory properties involving secretion of chemical mediators and migration of inflammatory cells. PD has been shown to suppress NO secretion and increase TNF-*α* in macrophages activated by lipopolysaccharide and interferon- (IFN-) *γ* [20]. In addition, PD suppresses the secretion of prostaglandin E2 (PGE2) in rat peritoneal macrophages stimulated by a protein kinase C activator [13, 20]. Seven-day pretreatment with PD showed significant inhibition of inflammation with a reduction in vascular permeability, leukocyte migration, and edema in RE model rats. In addition, antioxidant effects of PD were observed by a reduction in LPO and increases in the antioxidant enzymes SOD, CAT, and GSH. This suggests that PD downregulates NO synthesis, which may inhibit the production of free radicals, resulting in improvement of RE.

*Novel Drug Development Using Natural Herbal Products.* Currently, there has been an upsurge in research into developing novel drugs using herbal products [51]. PD contains five sugars identified as derivatives of oleanolic acid, a fatty acid. The number and types of sugar residues are thought to be involved in the diverse pharmacologic effects of PD. PD 3 is another single compound derived from Platycodon roots that is composed of six sugars and has less inhibitory effects on NO production and less stimulatory effects on TNF activity. In addition, while PD suppresses PGE2 production, PD 3 does not [13]. Another example is ginseng, which is composed of panaxadiol and panaxatriol with more than 10 derivatives [52]. Depending on the number of sugars, ginseng can have a variety of effects including antioxidant effects, anticancer properties, or immunomodulatory function [53]. Currently flourishing research has explored their therapeutic effects, and the chemical structures of novel drugs have been directly impacted by research into natural products. Therefore, further studies of pharmacological activity based on the structure of single compounds isolated from effective natural herbal products may provide important information for novel drug discovery.

## 5. Conclusion

The study revealed the therapeutic potential of PD regarding the suppression of histamine and gastric acid secretion accompanied by an enhancement of antioxidative system function in the gastrointestinal tract. PD has been used as a dietary supplement or medication in Korea with low potential for toxicity in humans, so further insights are expected from clinical trials designed to compare the efficacy of PD with other chemical compounds. This study provided important information, not only for RE therapeutics but also for drug discovery based on the structures of herbal compounds.

## Figures and Tables

**Table 1:** Effects of platycodin D on body weight in pretreatment groups.

Group	Body weight (g)		Changes (g)
	Day 0 1st pretreatment	Day 6 7th pretreatment	
Sham	176.4 ± 8.2	190.4 ± 7.0	14.0 ± 5.4
Control	177.4 ± 6.1	190.8 ± 10.6	13.4 ± 5.6
OMP	175.5 ± 7.5	189.6 ± 6.4	14.1 ± 4.6
PD 200	176.3 ± 9.9	189.5 ± 11.7	13.3 ± 6.8
PD 100	176.3 ± 8.7	190.9 ± 10.3	14.6 ± 4.3
PD 50	176.9 ± 10.5	189.6 ± 15.7	12.8 ± 6.5

The values are expressed as mean ± SD of eight rats. Changes were calculated according to (1) in Materials and Methods. PD: platycodin D, OMP: omeprazole.

**Table 2:** Effects of platycodin D on gastric secretion.

Group	Gastric secretion parameter		
	Gastric volume (ml/kg of body weight)	Gastric acids (*μ*Eq/6 hr)	Pepsin (*μ*mol/6 hr)
Control	11.5 ± 2.1	205.1 ± 17.1	927.6 ± 114.6
OMP	5.7 ± 1.0[−50.5]^*∗*^	95.3 ± 33.8[−53.6]^*∗*^	454.3 ± 211.0[−51.0]^*∗*^
PD 200	4.5 ± 1.4[−61.0]^*∗*^	74.3 ± 26.8[−63.8]^*∗*^	312.2 ± 101.1[−66.4]^*∗*^
PD 100	5.6 ± 1.2[−51.3]^*∗*^	92.3 ± 25.2[−55.0]^*∗*^	421.5 ± 126.4[−54.6]^*∗*^
PD 50	7.9 ± 1.7[−31.9]^*∗*^	123.2 ± 18.2[−39.9]^*∗*^	548.5 ± 102.1[−40.9]^*∗*^

Values are expressed as mean ± SD of eight rats. Data in [ ] mean percent changes compared to control (PCC). ^*∗*^*P* < 0.01 compared with control. PD: platycodin D, OMP: omeprazole.

**Table 3:** Antioxidant effects of platycodin D on gastric fundus.

Group	LPO (nM of MDA/mg protein)	Antioxidant defense system		
		SOD (U/mg protein)	CAT (U/mg protein)	GSH (mg/mg protein)
Sham	0.35 ± 0.17	96.30 ± 11.01	47.80 ± 8.51	45.54 ± 5.34
Control	1.17 ± 0.33^##^	44.73 ± 9.93^##^	20.44 ± 4.40^##^	18.46 ± 1.63^##^
OMP	0.77 ± 0.20[−34.5]^##,*∗*^	60.36 ± 12.10[34.9]^##,*∗*^	27.75 ± 3.21[35.8]^##,*∗*^	25.21 ± 4.54[36.6]^##,*∗∗*^
PD 200	0.60 ± 0.23[−48.6]^#,*∗∗*^	67.70 ± 19.04[51.4]^##,*∗∗*^	31.95 ± 5.92[56.3]^##,*∗∗*^	28.19 ± 5.19[52.7]^##,*∗∗*^
PD 100	0.74 ± 0.14[−36.6]^##,*∗∗*^	60.31 ± 9.98[34.8]^##,*∗*^	27.84 ± 4.69[36.2]^##,*∗*^	24.45 ± 4.21[34.1]^##,*∗∗*^
PD 50	0.87 ± 0.13[−25.8]^##^	55.58 ± 7.53[24.3]^##,*∗*^	26.07 ± 4.65[27.5]^##,*∗*^	23.40 ± 3.70[26.8]^##,*∗*^

Values are expressed as mean ± SD of eight rats; LPO: lipid peroxidation; MDA: malondialdehyde; SOD: superoxide dismutase; CAT: catalase; GSH: glutathione. Data in [ ] mean percent changes compared to control (PCC). PD: platycodin D, OMP: omeprazole. ^#^*P* < 0.05 and ^##^*P* < 0.01 compared with sham; ^*∗*^*P* < 0.05 and ^*∗∗*^*P* < 0.01 compared with control.

**Table 4:** Effects of platycodin D on gastric mucosal components.

Group	Gastric mucosal component		
	Total hexose (*μ*g/100 mg tissue)	Sialic acid (*μ*g/100 mg tissue)	Collagen (mg/100 mg tissue)
Sham	2675.67 ± 500.42	149.23 ± 15.16	18.72 ± 2.45
Control	1340.48 ± 202.74^#^	75.44 ± 16.03^#^	42.81 ± 5.93^#^
OMP	1946.79 ± 179.24[45.2]^#,*∗*^	109.91 ± 12.88[45.7]^#,*∗*^	28.08 ± 5.66[−34.4]^#,*∗*^
PD 200	2206.21 ± 350.50[64.6]^*∗*^	123.72 ± 10.50[64.0]^#,*∗*^	21.41 ± 3.36[−50.0]^*∗*^
PD 100	1943.86 ± 232.70[45.0]^#,*∗*^	108.28 ± 12.00[43.5]^#,*∗*^	28.52 ± 4.50[−33.4]^#,*∗*^
PD 50	1811.29 ± 235.21[35.1]^#,*∗*^	101.15 ± 7.91[34.1]^#,*∗*^	30.99 ± 4.76[−27.6]^#,*∗*^

Values are expressed as mean ± SD of eight rats. Data in [ ] mean percent changes compared to control (PCC). PD: platycodin D, OMP: omeprazole. ^#^*P* < 0.01 compared with sham; ^*∗*^*P* < 0.01 compared with control.

**Table 5:** Therapeutic effects of platycodin D on esophagus and fundus of stomach.

Group	Esophagus		Fundic stomach	
	Invasive lesion (%)	Thickness (*μ*m)	Invasive lesion (%)	Thickness (*μ*m)
Sham	1.42 ± 0.95	649.18 ± 139.75	3.87 ± 3.83	1802.52 ± 255.56
Control	83.62 ± 8.24^#^	1785.47 ± 269.89^#^	62.66 ± 7.31^#^	946.72 ± 161.64^#^
OMP	54.30 ± 8.41[−35.1]^#,*∗∗*^	939.09 ± 155.01[−45.0]^#,*∗∗*^	30.14 ± 3.70[−51.9]^#,*∗∗*^	1362.21 ± 273.10[43.4]^#,*∗∗*^
PD 200	40.73 ± 4.63[−51.3]^#,*∗∗*^	857.87 ± 189.94[−52.0]^*∗∗*^	12.55 ± 5.04[−80.0]^#,*∗∗*^	1554.51 ± 321.09[63.7]^*∗∗*^
PD 100	53.86 ± 9.93[−35.6]^#,*∗∗*^	988.39 ± 186.60[−44.6]^#,*∗∗*^	31.27 ± 8.27[−50.1]^#,*∗∗*^	1361.31 ± 300.32[43.3]^#,*∗∗*^
PD 50	68.03 ± 10.21[−18.6]^#,*∗*^	1189.89 ± 266.96[−33.3]^#,*∗∗*^	46.77 ± 6.91[−25.4]^#,*∗∗*^	1156.39 ± 114.53[21.8]^#^

Values are expressed as mean ± SD of eight rats. The percentage of invasive lesions and total thickness through the esophageal or gastric wall was measured using a microscopic analyzer. Data in [ ] mean percent changes compared to control. PD: platycodin D, OMP: omeprazole. ^#^*P* < 0.01 compared with sham; ^*∗*^*P* < 0.05 and ^*∗∗*^*P* < 0.01 compared with control.
